# COVID-19 Induced Myocarditis: A Rare Cause of Heart Failure

**DOI:** 10.7759/cureus.11690

**Published:** 2020-11-24

**Authors:** Qasim Z Iqbal, Muhammad Adnan Haider, Saud Bin Abdul Sattar, Muhammad Hanif, Ishaq Javid

**Affiliations:** 1 Internal Medicine, Northwell Health, New York City, USA; 2 Internal Medicine, Allama Iqbal Medical College/Jinnah Hospital, Lahore, PAK; 3 Internal Medicine, Khyber Medical College Peshawar, Peshawar, PAK; 4 Internal Medicine, Marshfield Clinic, Marshfield, USA

**Keywords:** covid 19, covid-induced myocarditis, heart failure with reduced ejection fraction, covid and myocarditis

## Abstract

Severe acute respiratory syndrome coronavirus 2 (SARS-CoV-2) causing lung injury has been well documented in the literature recently. They do so primarily by binding to the membrane-bound form of angiotensin-converting enzyme 2 (ACE-2) receptors. However, since these receptors are also expressed in the heart and blood vessels, coronavirus can also cause damage to these organs by binding to the ACE-2 receptors. A typical case of coronavirus disease 2019 (COVID-19) usually presents with respiratory symptoms like cough and shortness of breath accompanied by fever. The literature regarding this pandemic has been growing and now we know very well that the effect of this deadly virus is not restricted to the lungs alone. It can, unfortunately, cause various other complications ranging from neurological damage to even myocardial injury in rare cases. We present an interesting case of a 40-year-old male patient who presented to us with shortness of breath. When further investigated, the patient was found to have a new onset of heart failure secondary to COVID-19 induced myocarditis.

## Introduction

Coronavirus disease 2019 (COVID-19) has been found responsible for thousands of deaths all over the world. COVID-19 typically presents as fever, cough, fatigue, and headache. However, it has also been found to inflict neurological and myocardial damage on the patients [1]. Myocardial damage associated with COVID-19 has been found and recognized in recent literature [2]. These cardiovascular complications may include but are not restricted to myocarditis, myocardial infarction, dysrhythmias, and thromboembolic events. The first case of fulminant myocarditis secondary to COVID-19 was reported by Zeng et al. [3]. However, since this relatively rare complication can be easily missed by physicians, we emphasize with our case report the importance to keep a lookout for COVID-19 induced severe myocarditis in patients presenting with COVID-19 symptoms.

## Case presentation

A 40-year-old male with a past medical history of Type 2 diabetes mellitus presented to the ED with three days history of orthopnea, dyspnea, and productive cough. He presented to us during the peak of the COVID-19 pandemic and due to his characteristic symptoms, he was swabbed for the virus. The test came out positive. On his initial assessment, he had a rectal body temperature of 98°F, a systolic blood pressure of 90/50 mmHg, a heart rate of 114 beats per minute, a respiratory rate of 32 breaths per minute, and upon checking his pulse oximetry his oxygen saturation was found to be 82%. His physical examination was prominent for jugular vein distention, bilateral pedal edema, bi-basilar crepitations, and an S3 gallop. An initial diagnosis of acute heart failure was made and the patient was started on inotropic support and put on oxygen via nasal cannula.

His blood work was done (Table 1) and the investigations showed an elevation in his white blood cell (WBC) count, lactate dehydrogenase (LDH), C-reactive protein (CRP), blood urea nitrogen (BUN), creatine kinase, and troponin levels. The initial electrocardiogram (ECG) done showed sinus tachycardia. An echocardiogram (Table 2) was also done which revealed an ejection fraction of 21%-25% with moderate pulmonary hypertension with moderate mitral and tricuspid regurgitation. The patient was stabilized and taken for cardiac catheterization which showed no evidence of coronary artery occlusion. The patient was then taken for a cardiac MRI which revealed features of myocarditis (inflammatory hyperemia, edema, necrosis, contractile dysfunction, and accompanying pericardial effusion).

**Table 1:** Initial laboratory investigations. WBC, white blood cell; CRP, C-reactive protein; LDH, lactate dehydrogenase; BNP, brain natriuretic peptide; BUN, blood urea nitrogen

Test	Result
Hemoglobin	10.9 g/dL
WBC count	16.9 (x10^9^/L)
Red blood cell count	3.8 (x10^12^/L)
Platelets	370 (x10^9^/L)
Cardiac troponin I	7.3 ng/mL
Prothrombin time	15 s (12 s control)
Activated partial thromboplastin time	30 s (28 s control)
CRP	42 mg/Dl
LDH	701 U/L
Serum ferritin level	954.3 µg/L
BUN	52 mg/dL
Creatinine	1.1
BNP	1710 pg/mL
Creatine kinase	322 U/L

**Table 2:** Echocardiography findings. RVSP, right ventricular systolic pressure

Left atrial dimension	42 mm (normal range 19-40 mm)
Left ventricle end diastolic dimension	57 mm (normal range 36-56 mm)
Right ventricular dimension	30 mm (normal range 08-26 mm)
Fractional shortening	12%
Ejection fraction	21%-25% (normal range 55%-65%)
RVSP	58 mmHg

He was treated with dexamethasone, remdesevir, lasix, low doses of lisinopril, and metoprolol. His condition improved with the resolution of underlying viremia and he was discharged after 16 days of inpatient stay. He was advised to follow up with the cardiologist and primary medical doctor after two weeks.

## Discussion

The SARS-CoV-2 infection has been linked to a number of cardiovascular complications such as myocardial infarction, thromboembolic events, and myocarditis [1]. The first endomyocardial biopsy (EMB) proven case of myocardial inflammation in a COVID-19 patient was reported by Sala et al. [4].

The myocardial damage associated with SARS-CoV-2 infection has been proposed to be caused by:

1) Direct inoculation of the virus into the myocardium. SARS-CoV-2 uses its spike protein which is primed by transmembrane serine protease 2 (TMPRSS2) to bind to angiotensin-converting enzyme 2 (ACE-2) which facilitates cell entry. Thereafter, intracellularly, SARS-CoV-2 impairs the stress granule formation and without the stress granules, the virus replicates and damages the cell.

2) Immune response triggered by viral infection [5]. T lymphocytes are primed for viral antigens via antigen-presenting cells. The primed T lymphocytes migrate to the cardiomyocytes and trigger inflammation through cell-mediated cytotoxicity. The T lymphocyte activation is augmented in the cytokine storm syndrome, which results in a vicious positive feedback loop of immune activation and myocardial damage.

It is still not clear whether the pathogenesis of myocarditis is due to one of these mechanisms or both. Further studies are needed to provide clear information regarding it.

The clinical presentation varies; patients might present with mild symptoms such as excessive tiredness to in other cases presenting with acute heart failure. The diagnosis would require finding the presence of viral particles in the myocardium. The American Heart Association and European Society of Cardiology both currently recommend an EMB as the definitive diagnostic tool for myocarditis [6-7]. Furthermore, we have to rule out other similar clinical conditions based on a thorough history and clinical evidence. Investigations would include laboratory workup [inflammatory markers, cardiac enzymes, brain natriuretic peptide (BNP), and WBC], ECG, echocardiogram, cardiovascular MRI, and in some cases cardiac catheterization.

Our case shows a young patient without any comorbidities or previous heart disease who got a viral infection and developed myocardial inflammation within days [8]. Viral myocarditis in young adults is usually fulminant and hence unfortunately fatal in most cases [9]. Therefore, it is of utmost importance to distinguish fulminant myocarditis from a case of sepsis in the ER because fluid resuscitation would likely worsen the underlying fulminant myocarditis. Recent studies have further pointed out that myocarditis caused by coronavirus can present without the typical respiratory symptoms [10-11]. This only reinforces the importance of a detailed and in-depth history during the initial encounter with the patient in the ER.

## Conclusions

Myocardial damage due to COVID-19 has proven to be directly related to higher mortality in these patients. This is particularly true for myocarditis which can very easily cause a rapid deterioration in COVID-19 patients. SARS-CoV-2 damages the heart, by causing both structural and functional changes in the myocardium. Therefore, physicians should be extremely vigilant during this pandemic about the possibility of myocardial complications in COVID-19 patients as early diagnosis followed by accurate and precise management can potentially save the patient's life.
